# A Cost Analysis of Cardiac Magnetic Resonance Imaging in the Diagnostic Pathway of Patients Presenting With Unexplained Acute Myocardial Injury and Culprit-Free Coronary Angiography

**DOI:** 10.3389/fcvm.2021.749668

**Published:** 2021-10-20

**Authors:** Theodore Murphy, Daniel A. Jones, Rocco Friebel, Ijeoma Uchegbu, Saidi A. Mohiddin, Steffen E. Petersen

**Affiliations:** ^1^National Institute for Health Research (NIHR) Barts Biomedical Research Centre, William Harvey Research Institute, Queen Mary University of London, London, United Kingdom; ^2^Barts Heart Centre, St Bartholomew's Hospital, Barts Health National Health Service (NHS) Trust, London, United Kingdom; ^3^Department of Health Policy, London School of Economics and Political Science, London, United Kingdom

**Keywords:** cost-benefit analysis, cardiac magnetic resonance (CMR) imaging, myocardial infarction with non-obstructive coronary atherosclerosis, healthcare planning and management, financial modeling/forecasting

## Abstract

**Aims:** To determine financial implications of implementing cardiac magnetic resonance imaging (CMR) in the diagnostic pathway of a population with unexplained acute myocardial injury and normal coronary angiography.

**Methods and Results:** We performed a focused cost-benefit analysis using a hypothetical population of 2,000 patients with unexplained acute myocardial injury and normal coronary angiography divided into two groups to receive either standard or CMR guided management over a 10-year period. As healthcare practice and costs considerably vary geographically and over time, an algorithm with 15 key variables was developed to permit user-defined calculations of cost-benefit and other analyses. Using current UK costs, routine use of CMR increases healthcare spending by 14% per patient in the first year. After 7 years, CMR guided practice is cost neutral, reducing cost by 3% per patient 10 years following presentation. In addition, CMR -guided therapy results in 7 fewer myocardial infarctions and 14 fewer major bleeding events per 1,000 patients over a 10-year period. The three most sensitive variables were, in decreasing order, the cost of CMR, the cost of ticagrelor and the percentage of the population with MI requiring DAPT.

**Conclusion:** Routine use of CMR in patients with unexplained acute myocardial injury and normal coronary angiography is associated with cost reductions in the medium to long term. The initial higher cost of CMR is offset over time and delivers a more personalized and higher quality of care.

## Introduction

Acute presentations with myocardial injury that occur despite culprit-free coronary angiography were first reported over 70 years ago (1), with a prevalence of 5–9% in patients undergoing coronary angiography for suspected acute coronary syndromes (2–4). Historically, such presentations were often considered to represent myocardial infarction (MI) with non-obstructive coronary artery disease and referred to by the acronym “MINOCA.”

There is now considerable evidence that the majority of “MINOCA” presentations are not due to MI, and that a more accurate diagnosis is readily attainable in routine clinical practice following such presentations. In a meta-analysis of patients provisionally diagnosed with “MINOCA,” the use of cardiac magnetic resonance imaging (CMR) identified 33% had myocarditis, 24% had MI, 20% had other types of heart muscle disease (e.g., Takotsubo cardiomyopathy, dilated cardiomyopathy, or hypertrophic cardiomyopathy), and 26% had no CMR-detectable myocardial abnormalities (2). More recent studies corroborate these findings, emphasizing that MI is not the most frequent cause of this clinical presentation. These data also demonstrate a key role for CMR in the diagnostic pathway for patients with unexplained acute myocardial injury following culprit-free coronary angiography (5–8).

Due to the expectation that MI is the most frequent and/or important etiology of unexplained acute myocardial injury, guidance from the European Society of Cardiology (ESC) and American Heart Association (AHA) have historically focused on therapies for acute coronary syndromes in management following “MINOCA” presentations. In recognition of etiological heterogeneity of this acute presentation, recent guidelines focus on the role of improved diagnostic algorithms as key to improving patient outcomes (9–11). Within these algorithms is a consensus of opinion that CMR has a key role in the detection of the cause of the cardiac injury. However, it must be noted that as coronary artery disease remains the only frequent cause with therapies proven to improve outcomes, the certainty with which MI can be detected or excluded as the cause of acute myocardial injury remains the most important diagnostic aim at this time.

Moreover, while CMR's diagnostic role is supported by single center studies and meta-analyses (Class 1, Level of evidence B), there are no convincing economic evaluations of CMR in this setting (11). We aimed to model the financial impact of implementing CMR in the diagnostic pathway for assessing patients with unexplained acute myocardial injury and culprit-free coronary angiography.

## Methods

A hypothetical population of 2,000 patients with unexplained acute myocardial injury and normal coronary angiography was used for this modeling exercise. The population was split evenly into two groups receiving either standard management or CMR guided management. Each population was extrapolated over a 10-year period, with costs determined on an annual basis. Standard management was defined as 66% of the population receiving dual anti-platelet therapy (DAPT) for 1 year followed by aspirin for life, and the remaining 34% of the population receiving aspirin for life (3). CMR guided management ensured that only those with a “true” MI received DAPT for 1 year followed by aspirin for life.

Based on published data, 25% of the modeled population had a true MI (Table 1) (2, 5) and annual rates of a major bleed on DAPT, aspirin or no therapy were estimated as 2, 0.6, and 0.46%, respectively (2, 14–16). The assumed mortality rate in the first year after a diagnosis of unexplained acute myocardial injury and normal coronary angiography was 3.4 and 2.8% for subsequent years (3, 4). Expert consensus based on two seminal DAPT trials was used to determine the additional annual percentage likelihood of MI if not on DAPT and on aspirin only. This value was determined to be 2.6% (Table 1) (16, 17). Further details on the input values and the costs used are available in the Supplementary Material.

**Table 1:** Variables for modeling exercise.

**Cost ()**		
• Cost of major bleed	1,782	Weighted average (12)
• Cost of MI +Ticagrelor	2,188	Weighted average (12)
• Cost of CMR	385	Weighted average (12)
• Annual cost of Ticagrelor	710	(13)
• Annual cost of aspirin	10	(13)
• Inflation rate	3%	Assumed
**Rates**		
• Annual rate of major bleed on DAPT	2.20%	(2, 14)
• Annual rate of bleeding on aspirin	0.60%	Derived (15)
• Annual rate of bleeding on no agent	0.46%	Derived (16)
• Mortality rate in first year after diagnosis of unexplained acute myocardial injury and normal coronary angiography	3.40%	Derived (4)
• Mortality rate after first year of diagnosis of unexplained acute myocardial injury and normal coronary angiography	2.50%	Derived (3)
Additional % of repeat MI if not prescribed DAPT and prescribed aspirin only after a diagnosis of unexplained acute myocardial injury and normal coronary angiography	2.60%	Assumed (16)
% of Population with MI requiring DAPT	25%	Assumed (2, 5)

*DAPT, dual anti platelet therapy; MI, myocardial infarction; CMR, cardiovascular magnetic resonance imaging*.

However, despite only a fraction of patients having a true MI, the majority will be commenced on DAPT in clinical practice, as demonstrated in the largest registry of such patients where 66% received DAPT (3). Accordingly, the proportion of patients prescribed DAPT (66%) is higher than the assumed prevalence of MI (25%), and the majority of true MI's are likely to receive DAPT, leaving only a small proportion of true MI's at a relatively increased risk of future infarction.

A 10-year time horizon was selected for relevance to policy makers and because extrapolation beyond this would be subject to a high degree of uncertainty from changes in clinical practice and technology. A one-way sensitivity analysis was performed at year 10 to evaluate the variables entered into the model. An additional sensitivity analysis was performed by varying the proportion of patients placed on DAPT from 50 to 66%.

## Results

Fifty percent of the study population (1,000 individuals) were treated as per current practice and the remaining 1,000 individuals were treated within a CMR-guided pathway.

### Standard Practice

In the first year, the total cost is £507,402 for 1,000 patients, derived from the cost of ticagrelor to 66% of the population (£468,600); 15.24 major bleeding events (£27,158); aspirin for all (£10,000); and 0.75 MI's (£1,644). In the second and subsequent years, costs are significantly lower as ticagrelor and its risks for major bleeding are removed. Without adjusting for inflation, the second-year costs are from aspirin (£9,508), 5.70 major bleeding events (£10,166) and 0.74 MI's (£1,628). Over subsequent years, costs decrease incrementally as the population is censored.

### CMR Guided Practice

In the first year, the total cost is £580,058 for 1,000 patients, derived from the cost of 1,000 CMRs to 100% of the population (£358,000), ticagrelor to 25% of the population (£177,500); 8.45 major bleeding events (£15,058); aspirin for 25% (£2,500). In the second and subsequent years, costs are significantly lower as the cost of CMR is removed. Without adjusting for inflation, the second-year costs are from aspirin (£2,365), 4.74 major bleeding events (£8,439). Over subsequent years, costs decrease incrementally as the population is censored.

### Comparing Strategies

In the first year the CMR scans themselves account for two-thirds of the cost of the CMR guided practice. However, even in the first year, the costs of CMR (£385,000), are substantially offset by savings on ticagrelor (lower by £291,100), aspirin (£7,500), costs attributable to avoided major bleeds (£12,100), and those attributable to avoided MI (£1,644). When compared to standard practice, the CMR-guided pathway increases costs by 14% (£73) per patient in the first year.

**Figure 1:**
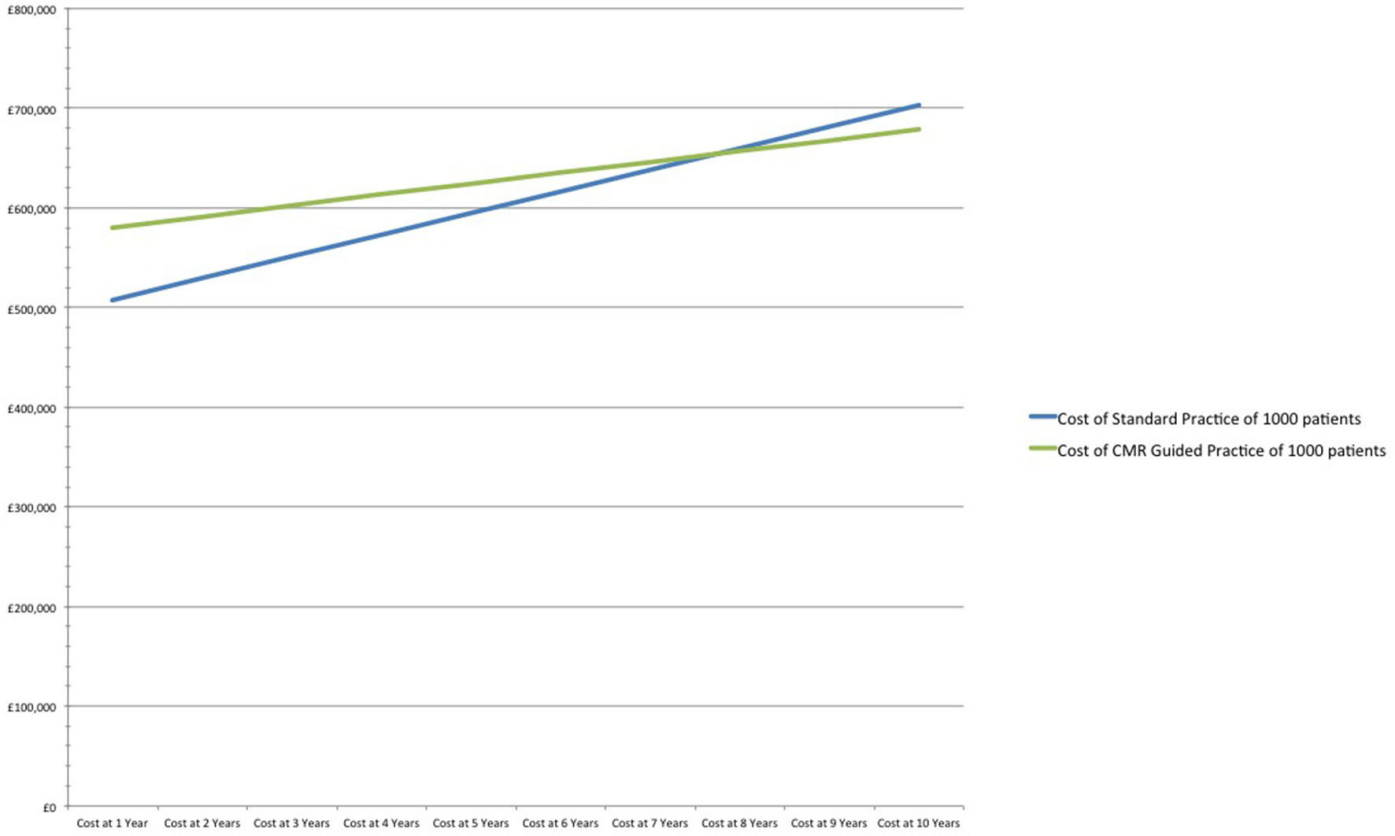
Cost analysis of standard practice vs. CMR guided practice at 66% DAPT.

After 7 years CMR guided practice becomes cost neutral, and at 10 years reduces costs by 3% (£23) per patient in comparison to standard practice. The relatively modest cost savings includes the prevention of 7 fewer MIs and 14 fewer major bleeds /1,000 patients over a 10-year period (Table 2 and Figure 1).

**Table 2:** Cost analysis of standard practice vs. CMR guided practice at 66% DAPT.

	**Cost at 1 year**	**Cost at 2 years**	**Cost at 3 years**	**Cost at 4 years**	**Cost at 5 years**	**Cost at 6 years**	**Cost at 7 years**	**Cost at 8 years**	**Cost at 9 years**	**Cost at 10 years**
Cost of standard practice of 1,000 patients	£507,402	£529,342	£551,215	£573,025	£594,771	£616,454	£638,077	£659,639	£681,143	£702,589
Cost of CMR guided practice of 1,000 patients	£580,058	£591,186	£602,268	£613,302	£624,290	£635,231	£646,126	£656,975	£667,778	£678,535
Cost difference CMR guided vs. standard practice	£72,656	£61,845	£51,052	£40,277	£29,519	£18,777	£8,049	–£2,664	–£13,365	–£24,054
Cost per patient (CMR guided vs. standard practice)	£73	£62	£51	£40	£30	£19	£8	–£3	–£13	–£24

*DAPT, dual anti platelet therapy; CMR, cardiovascular magnetic resonance imaging*.

### Sensitivity Analysis

**Figure 2:**
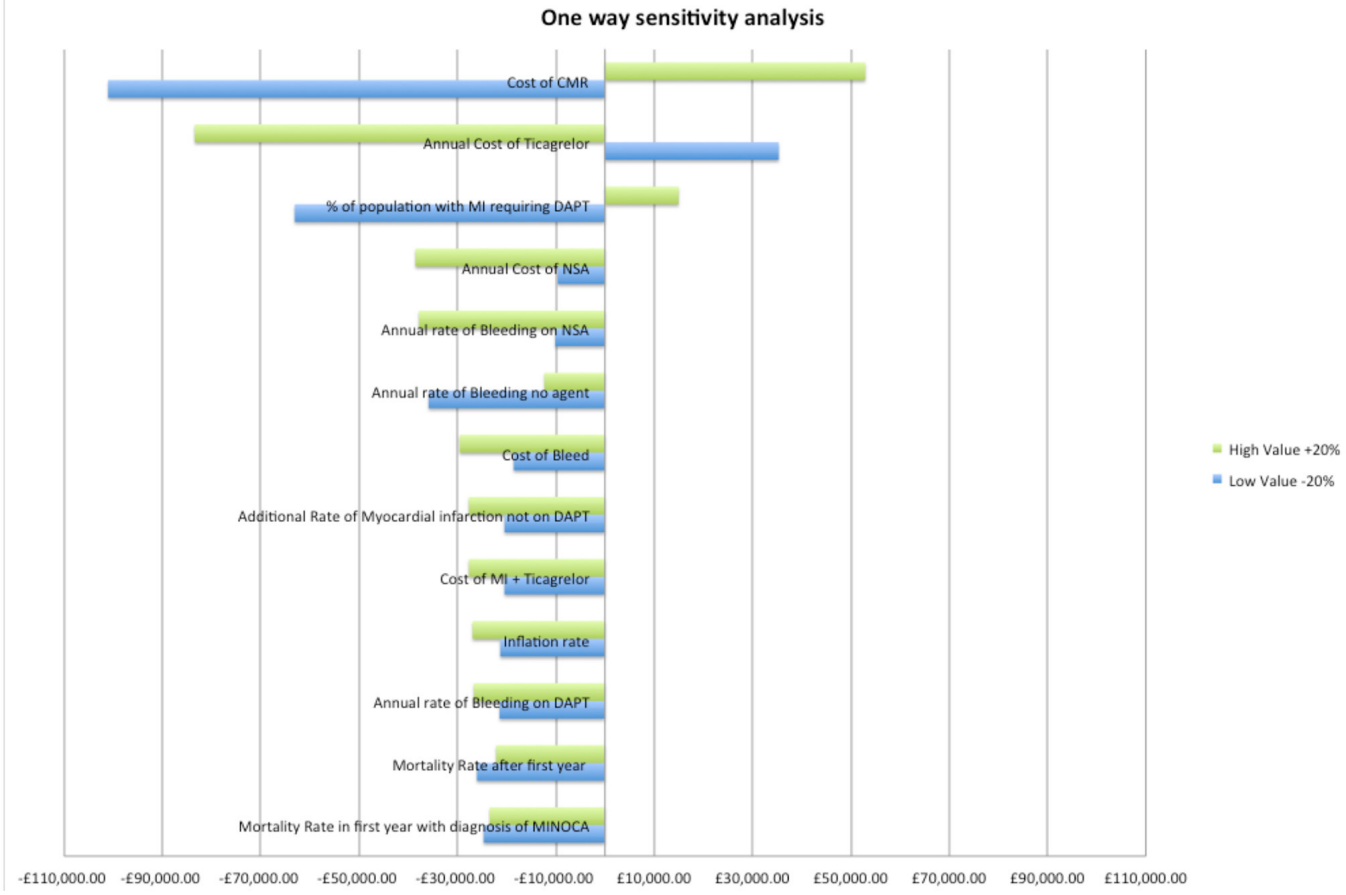
One-way sensitivity analysis of variables. DAPT, dual anti platelet therapy; MI, myocardial infarction; CMR, cardiovascular magnetic resonance imaging; NSA, aspirin.

A one-way sensitivity analysis determined the variables with largest impact on the costs. Each variable was varied by ±20% and the change in cost determined at year 10 (see Figure 2). The three most sensitive variables were, in decreasing order, CMR cost, the cost of ticagrelor and the frequency of true MI.

**Figure 3:**
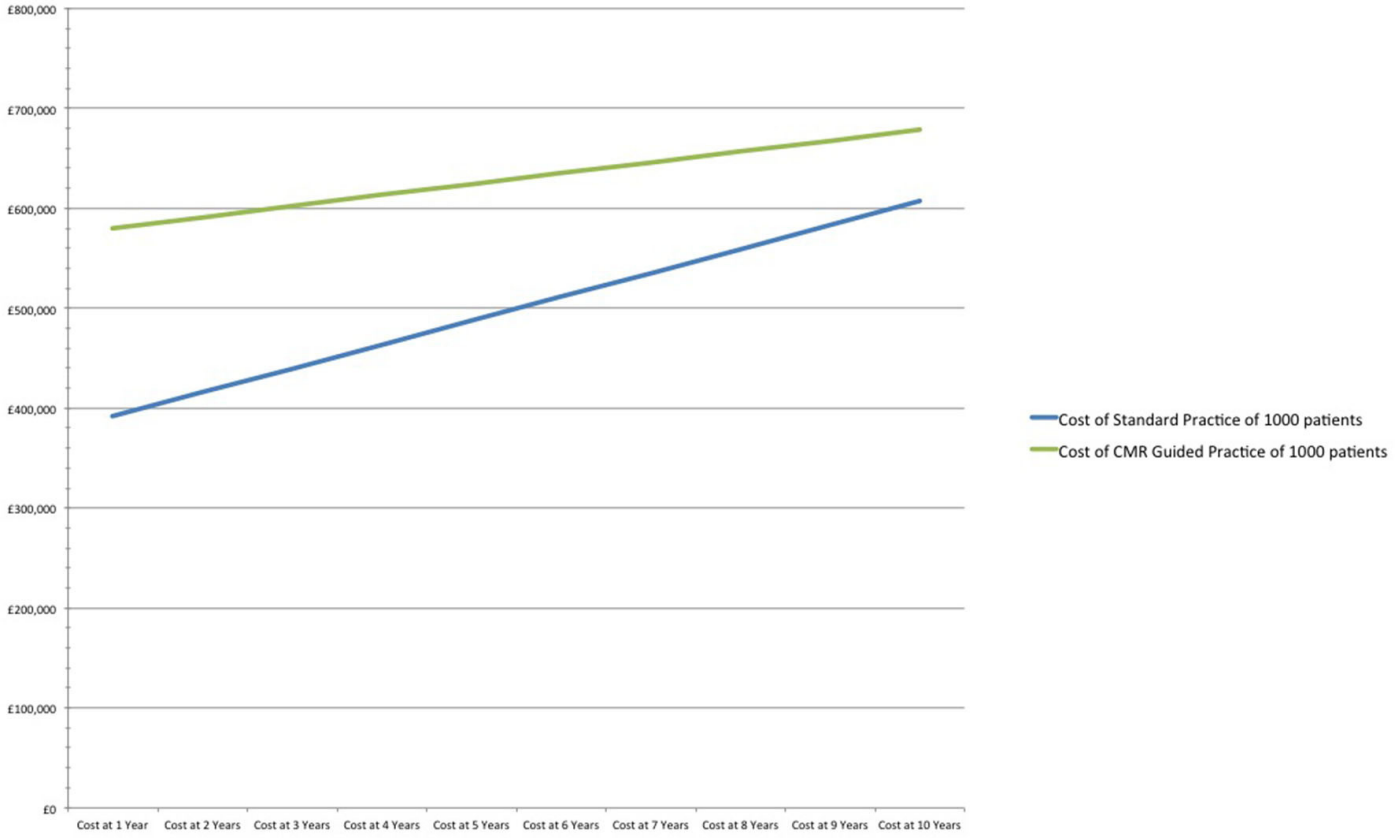
Cost analysis of standard practice vs. CMR guided practice at 50% DAPT.

If prescription rate of DAPT decreases from 66 to 50% in the standard practice arm, CMR's relative cost increases by 48% (£188/patient) in the first year when compared to standard practice. After 10-years, the CMR strategy remains 12% more expensive (£72/patient) in comparison to standard practice. However, CMR prevents 16 MIs and 12 major bleeds in the 10-year period (Table 3 and Figure 3).

**Table 3:** Cost analysis of standard practice vs. CMR guided practice at 50% DAPT.

	**Cost at 1 year**	**Cost at 2 years**	**Cost at 3 years**	**Cost at 4 years**	**Cost at 5 years**	**Cost at 6 years**	**Cost at 7 years**	**Cost at 8 years**	**Cost at 9 years**	**Cost at 10 years**
Cost of standard practice of 1,000 patients	£391,722	£415,670	£439,604	£463,527	£487,441	£511,349	£535,253	£559,156	£583,061	£606,970
Cost of CMR Guided practice of 1,000 patients	£580,058	£591,186	£602,268	£613,302	£624,290	£635,231	£646,126	£656,975	£667,778	£678,535
Cost difference CMR guided vs. standard practice	£188,336	£175,516	£162,663	£149,775	£136,849	£123,882	£110,873	£97,819	£84,717	£71,565
Cost per patient (CMR guided vs. standard practice)	£188	£176	£163	£150	£137	£124	£111	£98	£85	£72

*DAPT, dual anti platelet therapy; CMR, cardiovascular magnetic resonance imaging*.

If the prescription rate of DAPT was to increase to 100% in the standard practice arm, CMR is more cost effective with savings of £175,582 in the first year, and increased savings of £254,960 at year 10. Furthermore, using CMR prevents 19 major bleeds in the 10-year period.

An example equation used to calculate the costs associated with standard practice in Year 1 is available in the Supplementary File.

### Wider Perspectives

An estimation of the impact of nationwide adoption of CMR in the diagnostic pathway was based on 80,000 admissions with a diagnosis of presumed myocardial infarction in 2019/2020 (18) and with a 7% incidence of unexplained acute myocardial injury despite normal coronary angiography (~5,600 patients/year) (2–4). Routine use of CMR in the diagnostic pathway, when compared to standard practice, increases costs by £406,874 for year one. However, CMR use is associated with modest savings after 10 years (£138,226) and results in 42 fewer myocardial infarctions and 80 fewer major bleeding events.

## Discussion

Recent International guidelines have placed importance on the use of CMR in patients with unexplained acute myocardial injury and normal coronary angiography; however, the costs and benefits of adapting this approach remain poorly explored (10, 11).

**Figure 4:**
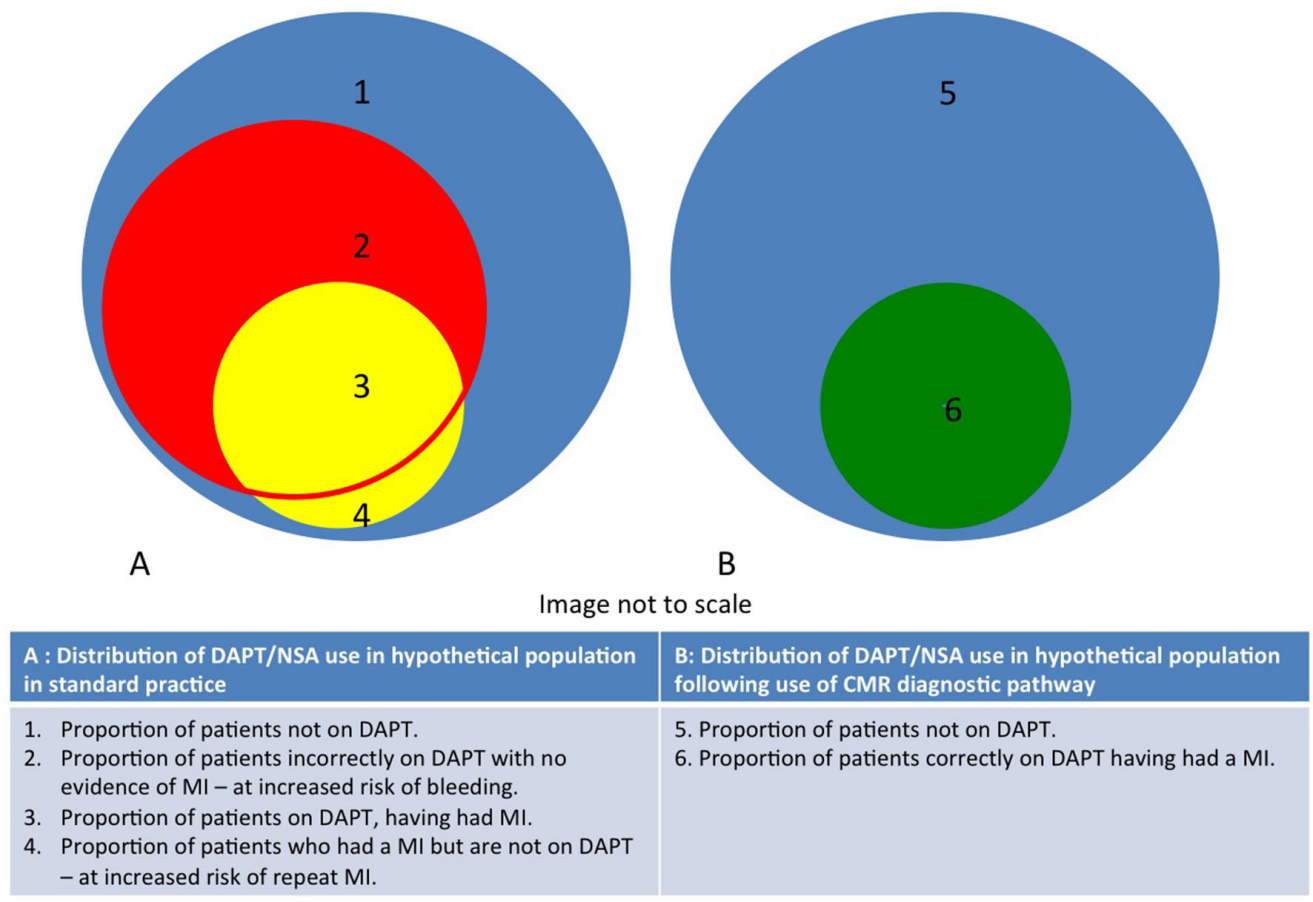
Diagram demonstrating distribution of DAPT/NSA use in populations with unexplained acute myocardial injury and normal coronary angiography in both CMR and standard practice. The routine use of CMR reduces the proportion of patients in areas 2 and 4.

Our analysis focuses on the benefits of identifying the fraction of such patients that have had a “true” MI: this group may benefit from secondary prevention, whilst those with alternative causes of myocardial injury are spared the costs of secondary prevention and its unwanted side effects (Figure 4). This relatively simple analysis demonstrates that CMR-guided management is relatively inexpensive in the short-term, may result in modest cost-savings in the medium to long term, and may reduce morbidity and mortality from major bleeding and recurrent MI.

A CMR guided strategy will also contribute to the identification of causes of cardiac injury other than MI (5, 11). It is important to note that healthcare and financial consequences of these other diagnoses are not included in the current analysis. Similarly, effect on quality of life, insurance premiums, critical illness status and several other ramifications following a diagnosis of MI have not been explored here. Although a more accurate diagnosis achieved with CMR may be expected to offer significant advantages in several of these domains, determining the attributable costs and benefits is uncertain; for example, the relevant financial implications of a diagnosis of myocarditis are undefined. Recognizing these challenges, our cost-benefit analysis necessarily focused on CMR's ability to accurately identify those patients who have had an MI in order to stratify accurately for antiplatelet therapy.

We only address treatment with DAPT and aspirin as other pharmaceutical agents commonly prescribed following MI (such as angiotensin converter enzyme inhibitors, angiotensin receptor blockers, beta-blockers and statins) are relatively inexpensive, have a low risk profiles, and include several agents commonly used for other cardiac conditions that present with acute cardiac injury. For the majority of these other causes of acute myocardial injury, the dearth of robust therapeutic data for the underlying diagnosis also limits our ability to estimate the effects of these agents on outcome (3, 19).

Within the constraints of a study limited to the benefits attributable to the identification of “true” MI, we identify the variables most sensitive to adjustment. These include the costs of CMR and ticagrelor, the percentage of the population with true MI, and the proportion of patients that would be prescribed DAPT in the absence of CMR stratification. In order to facilitate different healthcare practices, and account for cross-country variation in key determinants, our algorithm is available for download (Supplementary Material). This tool includes 15 adjustable variables to enable analyses to be better tailored to local factors and to accommodate changes in costs and in guidelines.

### Limitations

This analysis is limited by using a single study for determination of the proportion of missed myocardial infarctions and a single study for the determination of standard practice of DAPT usage (3, 8). In addition, the assumption of 100% sensitivity and specificity of CMR to diagnose infarction and the impact of false negatives, and false positives have not been included in the calculations and is an inherent limitation. This study is also restricted as it does not encompass the plethora of benefits that CMR has in this population, namely from a diagnostic, prognostic and management perspective. It is likely that the cost savings associated with CMR are underestimated as the timeline is only 10 years, and the population with unexplained acute myocardial injury with normal coronary angiography would likely be on aspirin for life, resulting in a greater number of bleeding events than reported in this study. Furthermore, the costs associated with MI are likely underestimated as this study did not account for the lifelong downstream costs, for example heart failure admissions and cardiac rehabilitation, again underestimating cost savings associated with CMR utilization. Although we have focused on invasive coronary angiography as the test that defines culprit-free coronary arteries, cardiac CT (and other techniques) may also determine culprit-free coronary status in patients presenting on ACS pathways. It is predicted that the use of CT will not alter this study's fundamental findings, except perhaps by identifying larger numbers of patients with cardiac injury where coronary disease is unlikely to be the cause and for whom CMR may help make important decisions for long-term management.

## Conclusion

This study, limited to an assessment of the utility of CMR to detect MI, demonstrates that the use of CMR in patients presenting with unexplained acute myocardial injury and normal coronary angiography includes modest short-term costs, is cost-reducing in the medium to long term, prevents treatment-related major bleeds, and reduces risks of subsequent MI.

This data advocates for the wider adoption of CMR, as its incorporation into acute cardiac pathways will help deliver a cost-effective, more personalized, and higher quality of care to patients.

## Data Availability Statement

The original contributions presented in the study are included in the article/Supplementary Material, further inquiries can be directed to the corresponding author.

## Author Contributions

TM, DJ, IU, SM, and SP contributed to conception and design of the study. TM created the calculator and wrote the first draft of the manuscript. TM, DJ, RF, and IU wrote sections of the manuscript. All authors contributed to manuscript revision, read, and approved the submitted version.

## Funding

This project was supported by the European Association of Cardiovascular Imaging (EACVI)—Task Force for Value Based Healthcare, and the British Heart Foundation accelerator award AA/18/5/34222.

## Conflict of Interest

TM acknowledges support from the Irish Cardiac Society. SP acknowledges support from the National Institute for Health Research (NIHR) Biomedical Research Centre at Barts. He provides consultancy to and is a shareholder of Circle Cardiovascular Imaging Inc., Calgary, Alberta, Canada. RF acknowledges support from AstraZeneca and Circle Cardiovascular Imaging Inc. These organizations were not involved in the study design, collection, analysis, interpretation of data, the writing of this article or the decision to submit it for publication. The remaining authors declare that the research was conducted in the absence of any commercial or financial relationships that could be construed as a potential conflict of interest.

## Publisher's Note

All claims expressed in this article are solely those of the authors and do not necessarily represent those of their affiliated organizations, or those of the publisher, the editors and the reviewers. Any product that may be evaluated in this article, or claim that may be made by its manufacturer, is not guaranteed or endorsed by the publisher.
