# Supplementary material for: A Cost Analysis of Cardiac Magnetic Resonance Imaging in the Diagnostic Pathway of Patients Presenting With Unexplained Acute Myocardial Injury and Culprit-Free Coronary Angiography
Source: Front Cardiovasc Med. 2021 Oct 20;8:749668. doi: 10.3389/fcvm.2021.749668 (PMC8564112; doi:10.3389/fcvm.2021.749668)
Supplement: Supplementary file 2 [file Data_Sheet_2.docx]

Supplementary information on costs, values and equations

**Input values for costing analysis:**

Costs were determined from a UK health care providers perspective.

Costs of a major bleed:

£ 1,782

Costs of a major bleed were determined from the perspective of the hospital, no further downstream costs were included.

Data was extracted from the NHS national reference costs from 2018-2019 available from: https://www.england.nhs.uk/national-cost-collection/

A weighted average of the costs associated with >120,000 patients admitted with gastrointestinal bleed, cerebrovascular hemorrhagic disorders and unspecified hematuria and their associated costs were calculated.

They were weighted accordingly: 50% gastrointestinal bleed, 35% cerebrovascular haemorrhagic disorders, 15% haematuria. This aims to reflect data from the ASCEND trial with a reported distribution of 41% GI bleeds, 21% ophthalmic bleeds, 17% intracranial bleeds, 20% other sites mainly haematuria. The costs were not available for ophthalmic bleeds, which is why the percentages were adjusted.

The costs were adjusted for inflation over the course of the 10-year time horizon.

Cost of myocardial infarction:

£2,188

Costs of a myocardial infarction were determined from the perspective of the hospital and the annual cost of ticagrelor, no further downstream costs were accounted for.

Data was extracted from the NHS national reference costs from 2018-2019 available from: https://www.england.nhs.uk/national-cost-collection/

A weighted average of the costs associated with >123,00 patients admitted with acute or suspected myocardial infarction was calculated, an additional cost of a one-year prescription of Ticagrelor was included. The costs were adjusted for inflation over the course of the 10-year time horizon.

Cost of CMR:

£385

Data was extracted from the NHS national reference costs from 2018-2019 available from https://www.england.nhs.uk/national-cost-collection/

Which provides a weighted average of >32,000 contrast CMR scans.

Medication cost:

£710 – Ticagrelor

£10 - Aspirin

Priced from NHS Business service authority drug tariff available on BNF 2020.

https://www.nhsbsa.nhs.uk/pharmacies-gp-practices-and-appliance-contractors/drug-tariff

Inflation rate:

3%

An arbitrary rate of 3% was selected.

Annual rate of major bleed on DAPT, aspirin or no therapy:

2% on DAPT, 0.6% on aspirin, 0.46% on no therapy

The rate of annual major bleed on DAPT with ticagrelor varies significantly depending on the population studied.

For example, the seminal PLATO trial reported 11.6% of the population had a major bleed in the first year with ticagrelor. ^[[1]](#endnote-1)^ In contrast, the more recent GLOBAL LEADERS study reported a 2-year bleeding rate of 2% for patients on DAPT (either ticagrelor or clopidogrel) for one year followed by aspirin for the second year.^[[2]](#endnote-2)^ The reported variation in the literature depends on the underlying population, but also the interpretation of what is considered a major bleed.

The authors of this report believe that a rate of 2% more appropriately reflects the MINOCA population. When bleeding values of 2% (DAPT) and 0.6% (aspirin) are utilized in a similar population as the largest registry of MINOCA patients – a 4-year bleeding rate of 3.2% over 4 years is achieved which although slightly lower correlates with their published results of 3.6% over 4.1 years. ^[[3]](#endnote-3)^

The bleeding rate for aspirin and no therapy was derived from ASCEND trial, which randomized ≈ 15,000 diabetic patients to receive aspirin or placebo with a mean follow up of 7.4 years. This trial resulted in 4.1% vs 3.2% bleeding rate over 7.4 years in aspirin and placebo arms, respectively. ^[[4]](#endnote-4)^

This trial was favored over the similar ARRIVE trial, which randomized ≈ 12,000 non-diabetic patients to receive aspirin or placebo and median follow-up of 5 years. This trial excluded patients at high risk of gastrointestinal bleeding and other bleeding, or diabetes, and only included GI bleeds and ICH resulting in a significantly lower event rate (0.97% vs 0.46%, aspirin vs placebo) compared with the ASCEND trial. ^[[5]](#endnote-5)^ In contrast, the ASCEND trial also included, sight-threatening bleeding event in the eye, or other serious bleeding.

The population was censored for death and major bleed at the end of each year.

Mortality rate in first year after MINOCA diagnosis:

3.4%

The mortality rate was determined from meta-analysis of >16,000 patients. ^[[6]](#endnote-6)^

This is below estimations that have placed mortality rates up to 16% at two years.^[[7]](#endnote-7)^

Mortality rate after first year of diagnosis of MINOCA:

2.8%

Based on data from the largest registry of MINOCA patients who were followed up for an average of 4.1 years, with a mortality rate of 13.4 % over 4.1 years. ^[[8]](#endnote-8)^

Using mortality of 3.4 % in the first year and 2.8 % in subsequent years, this equates to a comparable 4-year mortality of 11%, which approximates the 13.4% mortality rate reported in the literature.

Percentage of population with MI requiring DAPT:

25%

Several studies using CMR in MINOCA populations revealed that approximately 25% of the population had evidence of a MI recommending DAPT. ^3,^ ^[[9]](#endnote-9)^

Definition of standard Practice:

For the purposes of the analysis, standard practice was referenced from the largest reported MINOCA population documenting medication use. ^8^ The authors found that 66% of patients were placed on DAPT and over 90% on aspirin. The authors assumed similar figures, with 66% of patients on DAPT for one year followed by aspirin for life and the remaining 34% on aspirin.

Proportion of “missed myocardial infarctions”

25%

In a recent publication, 204 patients diagnosed with MINOCA were treated pre CMR, and then post CMR. In the pre CMR population 47 (23%) were misdiagnosed as not having had a myocardial infarction when CMR showed evidence that they had a MI. ^[[10]](#endnote-10)^ Similarly, this study assumed that 25% of myocardial infarctions were mis-diagnosed.

Despite this, being mis-diagnosed as not having had a MI, in clinical practice the majority of these patients will often be commenced on DAPT. This is reflected in the largest registry of MINOCA patients with a DAPT prescription rate of 66%. Based on this prescription rate, this would result in only 8% of the patients who had a MI not commencing on DAPT. ^8^

Note the percentage of patients who had an MI but were not on DAPT, changes depending on the % of population with myocardial infarction, and the % of patients being treated with DAPT.

Additional annual % likelihood of MI if not on DAPT and on ASPIRIN only:

2.6%

This figure was assumed by expert consensus from two seminal DAPT trials. The benefit of ticagrelor over clopidogrel was demonstrated in the PLATO trial with 5.8 vs 6.9 % patients developing repeat MI, resulting in a difference of 1.1%. ^1^ The benefit of using clopidogrel in addition to aspirin was demonstrated in the CURE trial with 5.2% vs 6.7% developing repeat MI, difference of 1.5%. ^[[11]](#endnote-11)^

Example equation –

*Cost of standard practice year 1:*

*Year 1:*

*DAPT population:*

*[Number of bleeds on DAPT] = Population x [% of patients on DAPT] x [Annual % of probability of bleed on DAPT]*

*[Number of deaths] = Population x [% of patients on DAPT] x [Annual % of death in 1^st^ year in MINOCA]*

*DAPT Population remaining to progress into year 2 = (Population x [% of patients on DAPT]) –([Number of bleeds on DAPT]+[Number of deaths])*

*NSA population:*

*[Number of bleeds on NSA] = Population x (1-[% of patients on DAPT]) x [Annual % of probability of bleed on NSA]*

*[Number of deaths in NSA population] = Population x (1-[% of patients on DAPT]) x [Annual % of death in 1^st^ year in MINOCA]*

*NSA Population remaining to progress into year 2 = (Population x (1-% of patients on DAPT)) –([Number of bleeds on NSA]+[Number of deaths in NSA population])*

*Avoidable MI*

*[Number of avoidable MI] = (Population x (1-[% of patients on DAPT])) x [Additional rate of myocardial infarction not on DAPT] x ((1-[% of patients on DAPT])/ (1/[% of population with MI requiring DAPT]))*

*Number of avoidable MI to progress into year 2 = (Population x (1-[% of patients on DAPT])) – (([Population x (1-[% of patients on DAPT])) x 1-mortality rate in 1^st^ year with diagnosis of MINOCA)*

*Cost year 1 =*

*[Cost of ticagrelor x (% of patients on DAPT)] + ([Cost of Aspirin]x Population) + (([number of bleeds on NSA] + [number of bleeds on DAPT])X [Cost of bleed]) + ([Number of avoidable MI]x ([cost of MI ]+ [cost of ticagrelor]))*

1. Wallentin L, Becker R, Budaj A, Cannon C, Emanuelsson H, Held C et al. Ticagrelor versus Clopidogrel in Patients with Acute Coronary Syndromes. New England Journal of Medicine. 2009;361(11):1045-1057. [↑](#endnote-ref-1)
2. Vranckx P, Valgimigli M, Jüni P, Hamm C, Steg P, Heg D et al. Ticagrelor plus aspirin for 1 month, followed by ticagrelor monotherapy for 23 months vs aspirin plus clopidogrel or ticagrelor for 12 months, followed by aspirin monotherapy for 12 months after implantation of a drug-eluting stent: a multicentre, open-label, randomised superiority trial. The Lancet. 2018;392(10151):940-949. [↑](#endnote-ref-2)
3. Pasupathy, S., Air, T., Dreyer, R., Tavella, R. and Beltrame, J., 2015. Systematic Review of Patients Presenting With Suspected Myocardial Infarction and Nonobstructive Coronary Arteries. *Circulation*, 131(10), pp.861-870. [↑](#endnote-ref-3)
4. The ASCEND Study Collaborative Group. Effects of Aspirin for Primary Prevention in Persons with Diabetes Mellitus. New England Journal of Medicine. 2018;379(16):1529-1539 [↑](#endnote-ref-4)
5. Gaziano, J., Brotons, C., Coppolecchia, R., Cricelli, C., Darius, H., Gorelick, P., Howard, G., Pearson, T., Rothwell, P., Ruilope, L., Tendera, M. and Tognoni, G., 2018. Use of aspirin to reduce risk of initial vascular events in patients at moderate risk of cardiovascular disease (ARRIVE): a randomised, double-blind, placebo-controlled trial. *The Lancet*, 392(10152), pp.1036-1046. [↑](#endnote-ref-5)
6. Pasupathy, S., Lindahl, B., Litwin, P., Tavella, R., Williams, M., Air, T., Marfella, R., Bainey, K., Alzuhairi, K., Reynolds, H., Johnston, N., Kerr, A. and Beltrame, J., 2019. 2389Survival after myocardial infarction with non-obstructive coronary arteries (MINOCA) - A comprehensive systematic review and meta-analysis. *European Heart Journal*, 40(Supplement_1). [↑](#endnote-ref-6)
7. Janosi, A., Ferenci, T. and Andreka, P., 2019. P878Prevalence and prognosis of patients with myocardial infarction with nonobstructive coronary arteries: a nationwide registry based study. *European Heart Journal*, 40(Supplement_1). [↑](#endnote-ref-7)
8. Lindahl, B., Baron, T., Erlinge, D., Hadziosmanovic, N., Nordenskjöld, A., Gard, A. and Jernberg, T., 2017. Medical Therapy for Secondary Prevention and Long-Term Outcome in Patients With Myocardial Infarction With Nonobstructive Coronary Artery Disease. *Circulation*, 135(16), pp.1481-1489. [↑](#endnote-ref-8)
9. Vágó, H., Szabó, L., Dohy, Z., Czimbalmos, C., Tóth, A., Suhai, F., Bárczi, G., Gyarmathy, V., Becker, D. and Merkely, B., 2020. Early cardiac magnetic resonance imaging in troponin-positive acute chest pain and non-obstructed coronary arteries. *Heart*, 106(13), pp.992-1000. [↑](#endnote-ref-9)
10. Dastidar, A., Rodrigues, J., Johnson, T., De Garate, E., Singhal, P., Baritussio, A., Scatteia, A., Strange, J., Nightingale, A., Angelini, G., Baumbach, A., Delgado, V. and Bucciarelli-Ducci, C., 2017. Myocardial Infarction With Nonobstructed Coronary Arteries. *JACC: Cardiovascular Imaging*, 10(10), pp.1204-1206. [↑](#endnote-ref-10)
11. Effects of Clopidogrel in Addition to Aspirin in Patients with Acute Coronary Syndromes without ST-Segment Elevation. New England Journal of Medicine. 2001;345(20):1506-1506 [↑](#endnote-ref-11)
